# Antioxidant Vitamins and Lipoperoxidation in Non-pregnant, Pregnant, and Gestational Diabetic Women: Erythrocytes Osmotic Fragility Profiles

**DOI:** 10.4021/jocmr454w

**Published:** 2010-11-19

**Authors:** Mohd Suhail, Shridhar Patil, Salma Khan, Sana Siddiqui

**Affiliations:** a Department of Biochemistry, University of Allahabad, Allahabad-211002, India; b City Nursing and Maternity Home Research Center, 21, Minhajpur, Allahabad-211003, India; c School of Life Sciences, Devi Ahilya University, Takshashila Campus, Khandwa Road, Indore-452 001, India; d Department of P.G. Studies and Research in Biological Sciences, RD University, Pachpedi, Jabalpur- 482001, India

## Abstract

**Background:**

Inconsistent reports are available in the literature regarding the oxidative status and antioxidant capacity during the pathogenesis of gestational diabetes. Present study was aimed to evaluate oxidative stress during the development of gestational diabetes and to evaluate antioxidant capability in non-pregnant (control), pregnant and gestational diabetics.

**Methods:**

The study consisted of non-pregnant, healthy pregnant and patients suffering from gestational diabetes mellitus (GDM). Each group consisted of 23 women. We compared their oxidative and anti-oxidative system in blood. Their blood malondialdehyde (MDA) and antioxidant vitamins (C, E, A) were determined and compared to evaluate the oxidative status and anti-oxidative capacity of these groups. We have also compared the osmotic fragility profiles of the erythrocytes of these groups.

**Results:**

Plasma MDA content in pregnant was significantly higher compared to non-pregnant (p < 0.001, 67.5%) and even in gestational diabetics; its value was found significantly further elevated (p = 0.001, 13.8%) compared to healthy pregnant. There was significant decline (p < 0.001, 41.9%) in the level of vitamin C in pregnant as compared to non-pregnant. Although in GDM the decrease was significant (p = 0.025, 20.6%) but comparatively lesser when compared to healthy pregnant. Vitamin E showed the increase of 9.6% during pregnancy, although this alteration was non-significant (p = 0.09), but the level was found to decline significantly (p < 0.001, 25.5%) in GDM compared to pregnant group. Vitamin A contents were also decreased in pregnant (p = 0.002, 17.4%) compared to non-pregnant and in GDM (p = 0.012, 11.2%) compared to pregnant group. Osmotic fragility (O.F.) profiles showed insignificant (p = 0.325) enhanced mean erythrocyte fragility (MEF) in pregnant but this increase was significant (p = 0.003) in case of GDM. The O.F. profiles of pregnant and GDM erythrocytes got shifted to the right side of the control one.

**Conclusions:**

Our findings indicate highly enhanced lipid peroxidation and significant depletion in antioxidant capacity during the development of gestational diabetes, and these alterations are not the cause but the consequence of GDM. However, further studies are warranted to examine a wider range of biochemical parameters to evaluate the potential risks of oxidative damage in GDM.

**Keywords:**

Gestational diabetes; Oxidative stress; Anti-oxidants; Vitamins C, E, A; Osmotic fragility; Non-pregnant; Pregnant; GDM

## Introduction

Gestational diabetes mellitus (GDM) is a heterogeneous disorder characterized by intolerance to carbohydrates and hyperglycemia in varied degrees of intensity, with onset or first diagnosis during pregnancy [1]. The pregnancy is a physiological situation of insulin resistance; therefore, it may be the first moment in a woman's life to test her capacity to respond to a physiological stress and to detect those at greater risk of developing diabetes in the future. Oxidative stress (OS) due to elevated reactive oxygen species (ROS) has been clearly linked to type 2 diabetes mellitus, however, limited and inconsistent data are available on the involvement of OS in GDM, a disease of similar pathophysiology. Women with GDM present a higher probability of developing higher risk markers of cardiovascular disease expressed as higher levels of lipid peroxides and descending levels of antioxidants. Further, that high blood glucose level induces oxidative stress and decreases antioxidant defenses. Fetuses born to mothers with gestational diabetes are at increased risk of developing respiratory distress, fetal macrosomia, fetal anomalies and platelet hyperaggregability. In a healthy body, ROS and antioxidants remain in balance. The OS occurs when this balance is shifted towards an overabundance of ROS. And OS results from an imbalance between pro-oxidants (free radical species) and the body's scavenging ability (antioxidants). The exact pro-oxidant and antioxidant status in gestational diabetes is still not clear. The present study addresses the possibility of lipoperoxidation and antioxidant activity in the blood of healthy non-pregnant, pregnant and gestational diabetic women.

A process that has been proposed as a common biochemical connection between chronic hyperglycemia and several physiologic functions relevant to diabetic complications is excessive non-enzymatic attachment of glucose to proteins [2]. Excessive formation of glycosylated proteins has been associated with alterations in the uptake of low-density lipoproteins [2] and with vascular damage mediated by the regulation of free radical formation [3]. Enhanced glycosylation by elevated glucose concentration may induce the formation of oxygen derived free radicals through protein glycosylation, which releases early and late glycosylated end products, contributing to enhancement of oxidative stress seen in diabetes [4]. Human erythrocytes are perhaps the cells most exposed to peroxidation damage by free radicals. The mechanisms by which the erythrocyte defends itself against oxidative damage are very efficient and are located in both cytosol and membrane domains. The membrane itself contains only vitamin E, the major lipid soluble chain breaking antioxidant [5].

Recent studies have examined the effect of intake of vitamins E and C, nutrients known to have antioxidant properties *in vivo*, on GHb concentrations in human subjects [6, 7]. Ceriello et al [6] found that large doses of tocopherol orally administered to individuals with diabetes lowered GHb concentrations. Davie et al [7] observed a similar pattern when large doses of ascorbic acid were orally administered to non-diabetic subjects. The mechanisms for this reduction in non-enzymatic glycosylation are not known but may be related to these nutrients' antioxidant properties or, for ascorbic acid, to a competition with glucose for protein binding. However, there is no consensus on the pathophysiological events underlying oxidative stress in diabetic patients, especially in pregnancy (GDM). Even, there is no consistency in the various reports regarding the levels of MDA contents and vitamins A, E and C in the plasma/blood of gestational diabetics as compared to the normal pregnant [8-15]. So far available data regarding prooxidant and antioxidant status in gestational diabetes are insufficient and controversial. In an earlier study, significant increase in lipid peroxidation level in diabetic pregnancy as compared to normal pregnancy was reported [16]. In contrast to this, an another study in early diabetic pregnancy found no evidence of greater lipid peroxidation in pregnant diabetics as compared to normal pregnant women, and total antioxidant capacity was also reported to be similar in both the groups [13].

Literature reveals very little information on erythrocyte osmotic fragility whereas this parameter is also significant to evaluate pathophysiological status of gestational diabetes. In 1987, increased osmotic fragility has been reported by us in the blood of diabetic patients which returned to normalcy after insulin treatment [17]. Our earlier report, that the enhanced oxidative stress results in increased osmotic fragility [18], tempted us to include osmotic fragility parameter also in the present study. Furthermore, there is a recent report in 2009 demonstrating that osmotic fragility of erythrocyte was greater in type 2 diabetic subjects compared to non-diabetic controls [19]. There are reports of no change in erythrocyte deformability [20, 21] whereas other have reported decreased deformability and increased red cell osmotic fragility in diabetics [17].

## Materials and Methods

### Chemicals

EDTA, Thiobarbituric acid (TBA) and butylated hydroxytoluene (BHT) were from Sigma Chemical Company (St. Louis, MO, USA). Other chemicals of analytical grade were obtained from E. Merck (Mumbai, India), BDH or SISCO Chemicals (Mumbai, India).

### Subjects

The patients in the present study included non-pregnant women (n = 23), normal healthy pregnant women (n = 23), gestational diabetic women (n = 23) admitted to the hospital who had been or not under regular care, and also those who were referred from private sectors or primary health centers. All the participants were within the age range of 21 - 32 years and the pregnant women were between 33 and 37 weeks of pregnancy. Body height and weight of the subjects were measured to calculate their body mass index (BMI).

Women were diagnosed with GDM if two or more of the 100 gram oral glucose tolerance test (OGTT) glucose levels exceeded the American Diabetes Association (ADA) criteria [22]: fasting: ≥ 95mg/dl (5.3 mmol/L); one hour: ≥ 180mg/dl (10.0 mmol/L); two hours: ≥ 155mg/dl (8.6 mmol/L) ; three hours: ≥ 140mg/dl (7.8 mmol/L). The present study was carried out with the prior approval of the local ethical committee. All the patients mentioned above gave their consent in writing, and the objectives of the study were fully explained to them in detail prior to taking consent. None of the participants had a family history of diabetes mellitus, hypertension, or obesity. Patients suffering from disease of any origin other than gestational diabetes were excluded from the study. Three groups were not having any additional supplement of vitamins.

### Sample collection

Blood samples were collected from the mothers at delivery. In each case, 10 mL blood were drawn into a sodium heparin vacutainer tube for separating plasma and stored at 4^o^C until processed. All samples were processed within 20 hours of sampling and plasma samples were stored at -70^o^C until required for vitamin analyses. Before storage, an equal volume of metaphosphoric acid (10%) was added to plasma samples designated for vitamin C analysis in order to deproteinize the plasma and stabilize the vitamin C content.

### Isolation of erythrocytes and hemolysate preparation

The blood samples were centrifuged at 1000 g for 15 min at 4^o^C and the isolated red cells were washed 4 - 5 times with 0.154 M NaCl to remove plasma and buffy coat. After final wash, the required packed red blood cells were lysed by hypotonic shock and different dilutions were used as hemolysate.

### Osmotic fragility protocol

Osmotic fragility (O.F.) experiments were performed following the method of Dacie and Lewis [23]. The NaCl concentration of 50% hemolysis was taken as a measure of mean erythrocyte fragility (MEF). Color measurement was made using Systronics colorimeter.

### Hemoglobin estimation

The method of Tentori and Salvati [24] was employed for hemoglobin estimation. Hemoglobin content of the sample was measured using cyanmethemoglobin method by mixing 20 μL of blood and 5 mL of (1:251 diluted) ferricyanide reagent [K_3_Fe(CN)_6_, 200 mg; KCN, 50 mg; K_2_HPO_4_, 140 mg; appropriate amount of detergent Triton X-100 dissolved and raised to one liter, pH 7.4] and allowing to stand for at least 3 min. Afterwards, absorbance was read at 540 nm using water as blank.

### Estimation of lipid peroxidation

Lipid peroxidation was quantified following the method of Jain et al [25]. Packed red cells (0.2 mL) were used for the quantification of malondialdehyde (MDA) as thiobarbituric acid reactive substances (TBARS). Aliquots of 0.2 mL were mixed thoroughly with 0.8 mL of phosphate-buffered saline (8.1 g NaCl, 2.302 g Na_2_HPO_4_, and 0.194 g NaH_2_PO_4_/L, pH 7.4) and 25 μL of butylatedhydroxytoluene (BHT, 88 mg/10 mL absolute alcohol) solution. After adding 0.5 mL of 30% trichloroacetic acid, the samples were vortexed and allowed to stand in ice for 2 h, and then centrifuged at 2000xg at 25^o^C for 15 min. One milliliter of supernatant was mixed with 75 μL of 0.1 M EDTA and 250 μL of 1% thiobarbituric acid in 0.05 M NaOH and placed on boiling water for 15 min. After cooling to room temperature, absorbance was measured at 532 nm and 600 nm. For evaluation of MDA, absorbance at 600 nm was subtracted from absorbance at 532 nm. BHT, an antioxidant, is added to prevent MDA formation during assay, which could result in falsely elevated TBA reactivity. The addition of BHT to standard MDA did not affect the color development with TBA. MDA content is expressed as nmol/gHb. Linearity established for MDA concentrations was ranging from 0.2 to 6 μmol/L, accuracy/recovery percentage was 90-95%, precision coefficient of variation (CV) values were 5% (intraday) and 12% (inter-days). LOD (limit of detection) and LOQ (limit of quantification) were 0.05 μmol/L and 0.09 μmol/L, respectively.

### Estimation of plasma vitamin C

Vitamin C concentrations were determined in plasma using the method of Jagota and Dani [26]. Plasma (0.2 mL) was precipitated on ice with 0.8 mL of 10% trichloroacetic acid for 5 minutes and then centrifuged at 1000xg for 5 min. A total of 0.5 mL of the supernatant was diluted with distilled water to the volume of 2 mL. Folin-Ciocalteau's solution (200 μL) diluted to 2 mL with distilled water, was added to the samples and mixed immediately. After 10 min, the absorbance at 760 nm of the blue color developed was measured spectrophotometrically. The sample values were compared with values of standard samples of ascorbic acid prepared in distilled water. Linearity established for vitamin C concentrations was ranging from 2 to 40 μg/mL, accuracy/recovery percentage was 93-100%, precision CV values were 2.5% (intraday) and 4.5% (inter-days). LOD and LOQ were 0.5 mg/L and 1 mg/L, respectively.

### Estimation of plasma vitamin A

Plasma vitamin A was estimated following the procedure of Sobel and Snow [27]. One milliliter of 95% ethanol was added to 1 mL plasma, the contents were mixed by tapping; 2 mL analytical reagent petroleum ether was added and the tube was shaken for 10 min. After shaking, the tube was centrifuged for about 30 seconds. The supernatant petroleum ether was aspirated and placed in a test tube. With another 2 mL of the petroleum ether, and shaking for only 5 min, the extraction procedure was repeated. The extract was evaporated to dryness by placing the tube in a 40-50^o^C water bath and running a stream of nitrogen over it. Analytical reagent grade chloroform (1 mL) was added to bring the dried extract into solution. Four milliliter of Glycerol dichlorohydrin (GDH) was added. The chloroform solution and the GDH were mixed and after 2 min, absorption was measured at 550 nm against a blank consisting of 4 mL of GDH and 1 mL of chloroform. Linearity established for vitamin A concentrations was ranging from 50 to 1200 μg/L, accuracy/recovery percentage was 87-96%, precision CV values were 7.5% (intraday) and 10.8% (inter-days). LOD and LOQ were 9 μg/L and 20 μg/L, respectively.

### Estimation of plasma vitamin E

A volume of 0.8 mL of plasma was pipetted into test tube and an equal volume of purified absolute ethanol was added to the tube for protein precipitation. The contents were immediately mixed with a vortex mixer, with 0.8 mL of xylene added, and the test tube was mixed for 30 s and centrifuged for 5 - 10 min at 1000xg. After centrifugation, the upper xylene layer containing extracted tocopherol was transferred to a small tube using a medicinal dropper. The tubes were covered with parafilm to avoid evaporation. Added 0.4 mL of plasma-xylene extract to the test tube containing 0.2 mL of 4, 7-diphenyl-1, 10-phenanthroline (bathophenanthroline, BA). A volume of 0.2 mL ferric chloride was added, followed by 0.2 mL of orthophosphoric acid. The contents of the tube were mixed thoroughly using a vortex mixer after every addition of reagents. The order of reagent addition is critical. Absorbance was read in the spectrophotometer at 536 nm after setting the instrument to zero absorbance with a blank (prepared by using 0.4 mL xylene instead of plasma-xylene extract). Linearity established for vitamin A concentrations was ranging from 0.8 to 20 mg/L, accuracy/recovery percentage was 85-90%, precision CV values were 6% (intraday) and 11% (inter-days). LOD and LOQ were 0.4 mg/L and 0.6 mg/L, respectively. Vitamin E contents were expressed as mol/L [28].

### Statistical analysis

SPSS version 15.0 for Windows (SPSS Inc., Chicago, IL, USA) software package was used to analyze the data of various parameters. The results were statistically analyzed using Student's t-test. The t-test statistical significance was set at P ≤ 0.05. Values were expressed as percentage and mean ± standard deviation.

## Results

The clinical characteristics of the different groups of patients are shown in Table 1. All the patients were within the age range of 21 - 32 years and were classified into three groups: Non-pregnant control group, pregnant group and gestational diabetics group.

**Table 1:** Clinical Profiles of the Patient Groups

Parameters	Non-pregnant Control Group [NP]	Pregnant Group [P]	Gestational Diabetics Group [GDM]	P^#^	P^# #^
Number of maternal	23	23	23		
Maternal age (years)	27 ± 4	26 ± 5	28 ± 4	0.46	0.14
Gestational age (weeks)	---	35 ± 1.6	35 ± 2.1	---	1.0
Maternal hemoglobin	12 ± 2.4	11 ± 2.2	11 ± 1.4	0.15	1.0
BMI at delivery ( kg/m^2^ )	20.1 ± 1.4	20.4 ± 2.6	21 ± 2.4	0.63	0.42
Birth weight (g)	---	2982 ± 389	3018 ± 423	---	0.77

Values are expressed as mean ± SD; BMI - body mass index; P = Two tailed probability; P^#^ = Comparison between NP - P ; P^# #^ = Comparison between P - GDM

**Figure 1.**
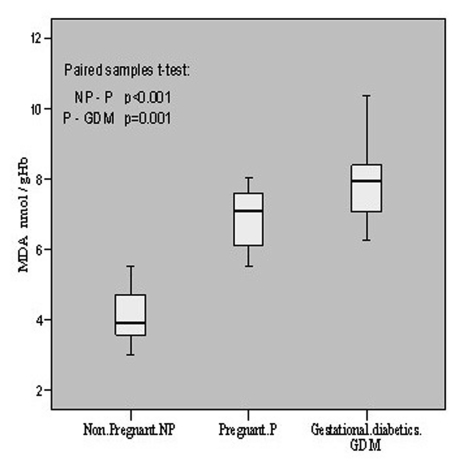
MDA contents in non-pregnant, pregnant, gestational diabetics.

The oxidative status was evaluated in terms of MDA contents in all the patients. In non-pregnant group it was 4.12 ± 0.76 nmol/gHb with a range of 3.02 - 5.54, in pregnant its mean content was 6.90 ± 0.82 having the range of 5.52 - 8.03 whereas in gestational diabetics the mean level was found to be 7.85 ± 0.92 which ranged from 6.25 to 10.36 nmol/gHb. Thus, the oxidative level in pregnant increased significantly (p < 0.001, t = -13.203) as compared to non-pregnant, and in gestational diabetics it also increased significantly (p = 0.001, t = -3.968) as compared to pregnant patients. These are summarized in Figure 1.

**Figure 2.**
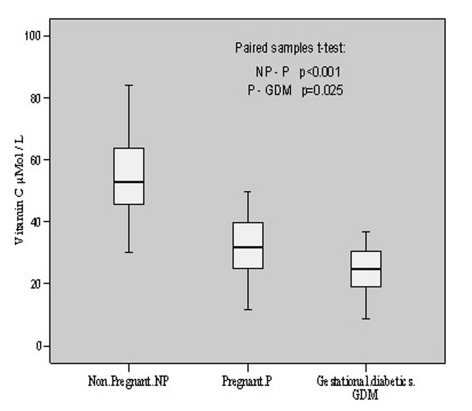
Vitamin C contents in non-pregnant, pregnant, gestational diabetics.

The anti-oxidative status of these individuals was estimated by evaluating the vitamins C, E and A levels. Vitamin C was found to be 54.26 ± 14.80 μmol/L in non-pregnant with individual content variation from 29.99 to 84.11 mol/L. In pregnant patients, its levels were found to vary from 11.80 to 49.54 with a mean value of 31.54 ± 11.07 μmol/L. However, in gestational diabetics its mean value was estimated to be 25.05 ± 7.63 μmol/L having individual value variations from 8.67 to 36.86 μmol/L. There was a decline (p < 0.001, t = 6.292) of vitamin C content in pregnant group as compared to non-pregnant. When gestational diabetics group was compared to normal pregnant group, the decrease was comparatively less (p = 0.025, t = 2.411). The overall alterations in vitamin C contents in these groups are shown in Figure 2.

**Figure 3.**
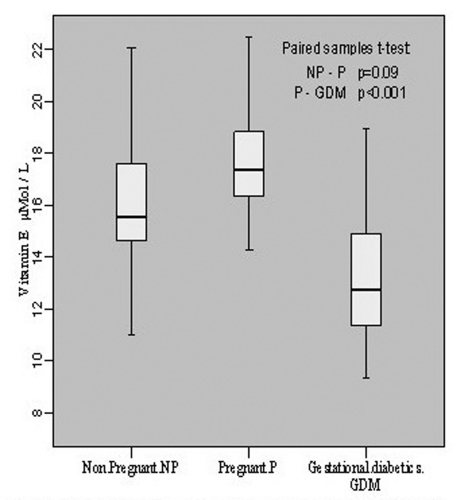
Vitamin E contents in non-pregnant, pregnant, gestational diabetics.

The estimation of vitamin E in non-pregnant group revealed its level to be 16.19 ± 2.92 μmol/L with individual level variations range from 11.02 to 22.08 μmol/L. In pregnant group, the mean value was 17.74 ± 2.16 with individual levels ranging from 14.29 to 22.48 μmol/L whereas in gestational diabetics the mean level was 13.21 ± 2.64 mol/L of the individual values ranging from 9.32 to 18.98 μmol/L. This showed an insignificant increase (p = 0.09, t = -1.774) in vitamin E content during pregnancy when compared with non-pregnant group, however, the decline in vitamin E level was significant (p < 0.001, t = -5.793) in gestational diabetics as compared to normal pregnant group (Fig. 3).

**Figure 4.**
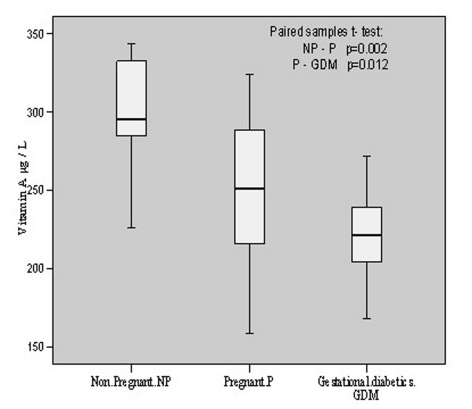
Vitamin A contents in non-pregnant, pregnant, gestational diabetics.

Vitamin A content in non-pregnant group was found to be 302.08 ± 35.48 μg/L with the individual contents varying from 225.63 to 343.79 μg/L. Its level in pregnant group was 249.53 ± 49.00 μg/L with the variation of 158.68 - 324.22 g/L contents in the individual pregnant patient. In gestational diabetics, the mean value was found to be 221.50 ± 26.44 having individual variation range of 167.65 - 271.84 μg/L. Thus, there was significant (p = 0.002, t = 3.509) decrease of vitamin A contents in pregnant group in comparison to non-pregnant group whereas this decline was comparatively less (p = 0.012, t = 2.728) in gestational diabetics as compared to pregnant group. These changes have been depicted in Figure 4.

**Figure 5.**
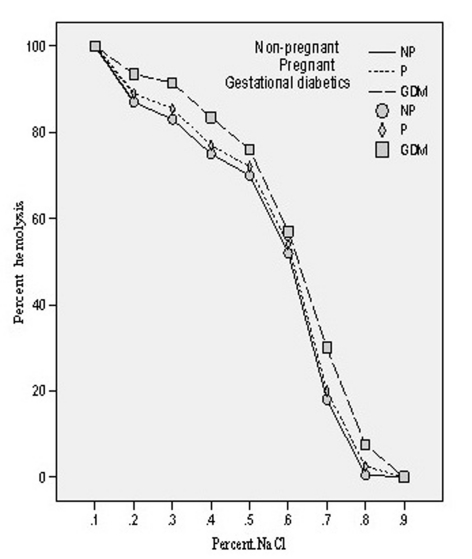
Osmotic fragility profiles of the erythrocytes of non-pregnant, pregnant, gestational diabetics.

The osmotic fragility of the mixed population of red blood cells obtained from all the three groups of patients was measured by scoring hemolysis in hypo-osmolar solutions (0.10-0.90% NaCl). The osmotic fragility (O.F.) profile of pregnant and gestational diabetics showed shift to the right side which shows that O.F. got increased in both the cases. The concentrations of saline solution causing 50% of hemolysis in all the three groups have been evaluated and designated as Mean Erythrocytes Fragility (MEF). In case of non-pregnant group, it was found to be 0.61 ± 0.018 whereas in pregnant and gestational diabetics, these were 0.615 ± 0.016 and 0.628 ± 0.012. The enhancement of MEF was insignificant (p = 0.325, t = -1.00) in case of pregnant compared to non-pregnant whereas in gestational diabetics the increase was found to be significant (p = 0.003, t = -3.12). The osmotic fragility profiles of three groups are depicted in Figure 5.

## Discussion

Gestational diabetes mellitus is a heterogeneous disorder characterized by intolerance to carbohydrates and hyperglycemia in varied degrees of intensity, with onset or first diagnosis during pregnancy. The pregnancy is a physiological situation of insulin resistance; therefore, it may be the first moment in a woman's life to test her capacity to respond to a physiological stress and to detect those at greater risk of developing diabetes in the future. Direct measurement of free radicals is difficult due to their unstable and transient nature; therefore, the tendency of free radicals to cause lipid peroxidation has been used as an indirect measure. In the present study the marker of lipid peroxidation (MDA) was found to increase during the progression of normal pregnancy. An increase of 67.5% MDA content in pregnant group as compared to non-pregnant group, further elevation of 13.8% in GDM-group as compared to pregnant group, and overall increase of 90.5% in GDM group in comparison with non-pregnant healthy group were observed. Such alteration suggests an easier membrane lipoperoxidability and, consequently, easier membrane damage during diabetic gestation.

In diabetes mellitus, increased blood glucose levels induce oxidative stress. Possible source of oxidative stress and damage to protein in diabetes include free radicals generated by auto oxidation of unsaturated lipids in plasma and membrane proteins [29]. Our observation of increased osmotic fragility followed by significant enhancement of MDA level in gestational diabetics (GDM) is in harmony with the report of Spickett et al [30]. Furthermore, increased osmotic fragility results from oxidative damage to the erythrocyte membrane, causing a decrease in membrane fluidity and reducing its ability to withstand osmotic changes. We have previously reported increased osmotic fragility of diabetic erythrocytes which on insulin treatment gave normal osmotic fragility profile [17]. The main determination of *in vitro* hemolysis is the volume of the cell at any given time in relation to its maximal possible membrane surface area. *In vitro* osmotic fragility is dependent on: (1) the suspending medium, whose pH and tonicity are controlled in the osmotic fragility test; (2) total number of intracellular osmotically active constituents, which determine cell volume in any given external environment; and (3) the critical hemolytic volume, a complex parameter dependent on quantitative and qualitative factors associated with the membrane lipid and protein. Therefore, the important relationship determining osmotic fragility is the ratio of critical hemolytic volume to the internal osmotic contents of the red blood cell. Our results on the red cells contents of MDA in normal and GDM clearly showed a significant change in their internal contents. Significant increase in the O.F. in GDM would ultimately results in decreased deformability of their erythrocytes leading to their shortened lifespan.

Vitamin E is a chain breaking antioxidant protecting the lipid phase of the cell from oxidative chain reactions [31, 5] and is an important lipid-soluble antioxidant in human plasma. The antioxidant effect of tocopherols is mainly due to their ability to donate hydrogens from the phenolic ring of the molecule to lipid radicals [32]. A tocopheroxyl-radical is then formed that can be reduced back to tocopherol.

Antioxidant vitamins, with the ability to stabilize highly reactive free radicals, act as the first line of defense against free radical attack and lipid peroxidation. Vitamins E (α-tocopherol) and C, have differences in the contribution they make to antioxidant potential, as vitamin E is the major lipid soluble chain-breaking antioxidant in cell-membranes while vitamin C is an important aqueous phase antioxidant. Antioxidants may act synergistically, for instance, when vitamin C regenerates α-tocopherol from the tocopherol radical, this 'sacrificial' antioxidant acts more by sparing vitamin E than by recycling. The important role of vitamin C in gestational diabetes, suggests that changes in its concentration may influence susceptibility of vascular endothelium to oxygen toxicity. Thus, our present study on vitamin C concentration may provide a means of assessing the total capacity of the chain-breaking antioxidants to prevent lipid peroxidation in plasma and it might be important to evaluate the effectiveness of potential antioxidant defense systems in limiting scale. This study, further, provides evidence for the relationship between plasma vitamin C levels during the pregnancy and gestational diabetes. Our results showed 53.8% decrease in vitamin C concentration in gestational diabetics as compared to non-pregnant and 20.6% decrease as compared to normal healthy pregnant. Our observation of such significant decline in vitamin C concentration is consistent with the reports [14, 15] but contrary to those [13, 9] who have reported no change in vitamin C contents in GDM.

Both vitamins C and E behave as scavengers of ROS and interestingly, our results showed an increase of 9.6% vitamin E level during pregnancy. During normal pregnancy, plasma vitamin E concentrations show a progressive elevation, what could be due to the gestational increase in circulating lipoproteins, the transporters of vitamin E.

However, its level declined to the extent of 25.5% in GDM in comparison to pregnant and 18.4% as compared to non-pregnant. Our finding on such significant decrease of vitamin E in GDM is in harmony with those reported earlier [10, 11, 14] and contrary to others [8, 12] who have reported increase in vitamin E level during the development of GDM. Another antioxidant, vitamin A was also found to decrease in its contents significantly; 11.2% and 26.7% as compared to pregnant and non-pregnant respectively, which is consonant with the report [10] but different from others [13] who reported no change in its level in GDM.

Thus, our findings indicate highly enhanced lipid peroxidation and significant depletion in antioxidant capacity during the development of gestational diabetes and these alterations are not the cause but the consequences of GDM. Our data reveal that antioxidant defense mechanisms might be impaired in patients with gestational diabetes.

These variations suggest an easier membrane lipoperoxidability and, consequently, an easier RBC membrane damage resulting in their shortened life-span, during diabetic gestation. The increased oxidative stress we found in pregnant women with GDM should be monitored by strictly controlling blood glucose during pregnancy with stringent recommendations and perhaps antioxidant supplementation. However, further studies are warranted to examine a wider range of biochemical parameters to evaluate the potential risks of oxidative damage in GDM.
